# Implementation of a Resident Pod Associated With Increased Patient Encounters and Critical Procedures for Emergency Medicine Residents in a Community ED

**DOI:** 10.7759/cureus.101018

**Published:** 2026-01-07

**Authors:** Jamie Lam, Hanna Rahman, Morah D Brown, Nathan D Stuempfig

**Affiliations:** 1 Emergency Medicine, Kaiser Permanente Central Valley, Modesto, USA; 2 Biostatistics, Kaiser Permanente, Pleasanton, USA

**Keywords:** critical care, emergency department, emergency medicine procedures, emergency medicine residents, medical education

## Abstract

Background

Currently, there are varied clinical workflows throughout Emergency Medicine (EM) training programs, without recommendations that optimize resident learning opportunities. Furthermore, newer, community-based programs often have difficulty integrating residents into existing workflows. It is critical for EM training programs to optimize opportunities to perform advanced, critical procedures and to provide adequate patient volumes for their residents.

Methods

This is a retrospective, observational study conducted in a single, community-based ED. Data were collected for 1 year prior to the implementation of a Residency Pod (R Pod) and for 1 year after implementation. Postgraduate Year (PGY)-1 and PGY-2 classes were used in each data set. There were eight residents in each class, for a total of 16 residents for each timeframe. The median number of patients seen per month, as well as critical procedures per month, was calculated. The Wilcoxon rank-sum test was utilized to determine statistical significance.

Results

There was a statistically significant increase in both patient encounters per month and critical procedures performed by residents per month. For patient encounters, statistical significance was obtained for the PGY-1 residents (p=0.004) and for all residents (p=0.022). Procedures increased for PGY-1s (p=0.002), PGY-2s (p=0.041), and all residents (p=0.002). PGY-2 residents saw more patients in the R Pod, but this did not achieve statistical significance.

Conclusion

The creation and implementation of an R Pod resulted in increased patient volumes and increased opportunities to perform critical procedures for EM residents when compared to a round-robin patient assignment system. Although this is a small, single-center study, consideration of utilizing an R Pod clinical structure may be warranted for new, community-based EM residency programs.

## Introduction

Emergency Medicine (EM) residency programs are pivotal in shaping the next generation of physicians capable of delivering critical care under pressure. EM residency training programs emphasize a blend of practical experience and theoretical knowledge, ensuring that residents are well-prepared to handle the unpredictable nature of emergency care. Through a structured curriculum that includes direct patient care, simulation training, and didactic learning, residents are equipped to deliver high-quality emergency care and respond adeptly to the diverse challenges that arise in this fast-paced field. The cornerstone of EM residency training programs is the exposure to, and experience with, a sufficient number of patient clinical encounters. Patient acuity and procedural volume are also critical components of EM residency training [1]. Because of this, properly balancing the number of residents per patient volume is crucial. The American Academy of Emergency Medicine (AAEM) recommends a standard of one resident per 3,600 patients at the primary training site to ensure adequate volumes of patient exposure, procedural opportunity, and educational quality [2].

There are multiple ways in which EM residency programs implement their hands-on direct patient care, and there is a spectrum that these stylistic differences fall into [3]. Some residencies have residents work 1-on-1 with attending physicians, and at the other end of the spectrum, some residencies have residents in charge of the entire ED with minimal input from the attending physician. A descriptive analysis revealed that residents' patient volume increased progressively with each year of training, with Post Graduate Year (PGY)-1, PGY-2, and PGY-3 residents seeing an average of 0.81, 1.05, and 1.27 patients per hour, respectively [4]. Additionally, research suggests that resident productivity is a dynamic process, which should be considered in staffing decisions and studied further [5]. Also, it is important to note that previous studies indicate the implementation of an EM residency program led to a 70% increase in attending physician productivity, measured in relative value units (RVUs) per hour [6]. This allows attending physicians to evaluate a larger number of patients if they are supervising residents compared to treating patients themselves.

In July 2021, Kaiser Permanente Northern California (KPNC) Modesto Medical Center launched a three-year, community-based EM training program with a complement size of 8 residents per year. Due to the high volume of daily patients, smaller program size, off-service rotation commitments, and Accreditation Council for Graduate Medical Education (ACGME) duty hour requirements, it was not possible for residents to care for every ED patient. Initially, the resident shifts were designed to be 1-on-1 with attending physicians. The attending physicians were assigned patients in a cyclical, sequential rotation, and residents would see these patients as they were assigned and roomed. Patient acuity was not factored into the assignments, and critically ill patients were often assigned to attending physicians not working with residents. When the third cohort joined in July 2023, resident and attending physician feedback compiled by the Program Evaluation Committee (PEC) indicated concerns that residents were not receiving enough exposure to critical patients or procedures. In addition, there were concerns that residents were not having sufficient autonomy of their patients and that residents were not developing enough skill set to gain a global awareness of the department.

To address these concerns, a new model called the Residency Pod (R pod) system was introduced for all PGY classes at the beginning of the 2023 academic year, which sought to ensure supervisory learning at every level, not just from attending physicians but from senior residents as well, offering a valuable educational experience for residents [7]. In addition, literature suggests that early assignment of patients to specific treatment teams, which is incorporated by the R pod assignment system, improves length of stay, patient satisfaction, resident education, and decreases the rate of patients leaving without being seen [3,6,8]. The primary aim of this study is to evaluate the impact of the R pod system on overall patient volume, critical procedure volume, and exposure to high-acuity patients for EM trainees.

This article was previously presented as an abstract at the Council for Residency Directors in Emergency Medicine Academic Assembly on March 3rd, 2025, the AAEM Scientific Assembly on April 7th, 2025, and the Society for Academic Emergency Medicine Annual Meeting on May 16th, 2025.

## Materials and methods

System model description

**Figure 1:**
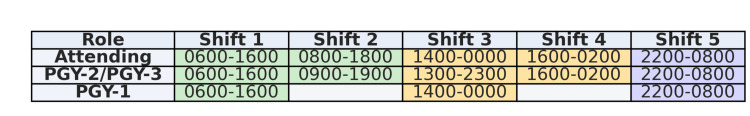
Sample R pod staffing model. R Pod: Residency Pod; PGY: Postgraduate Year.

**Figure 2:**
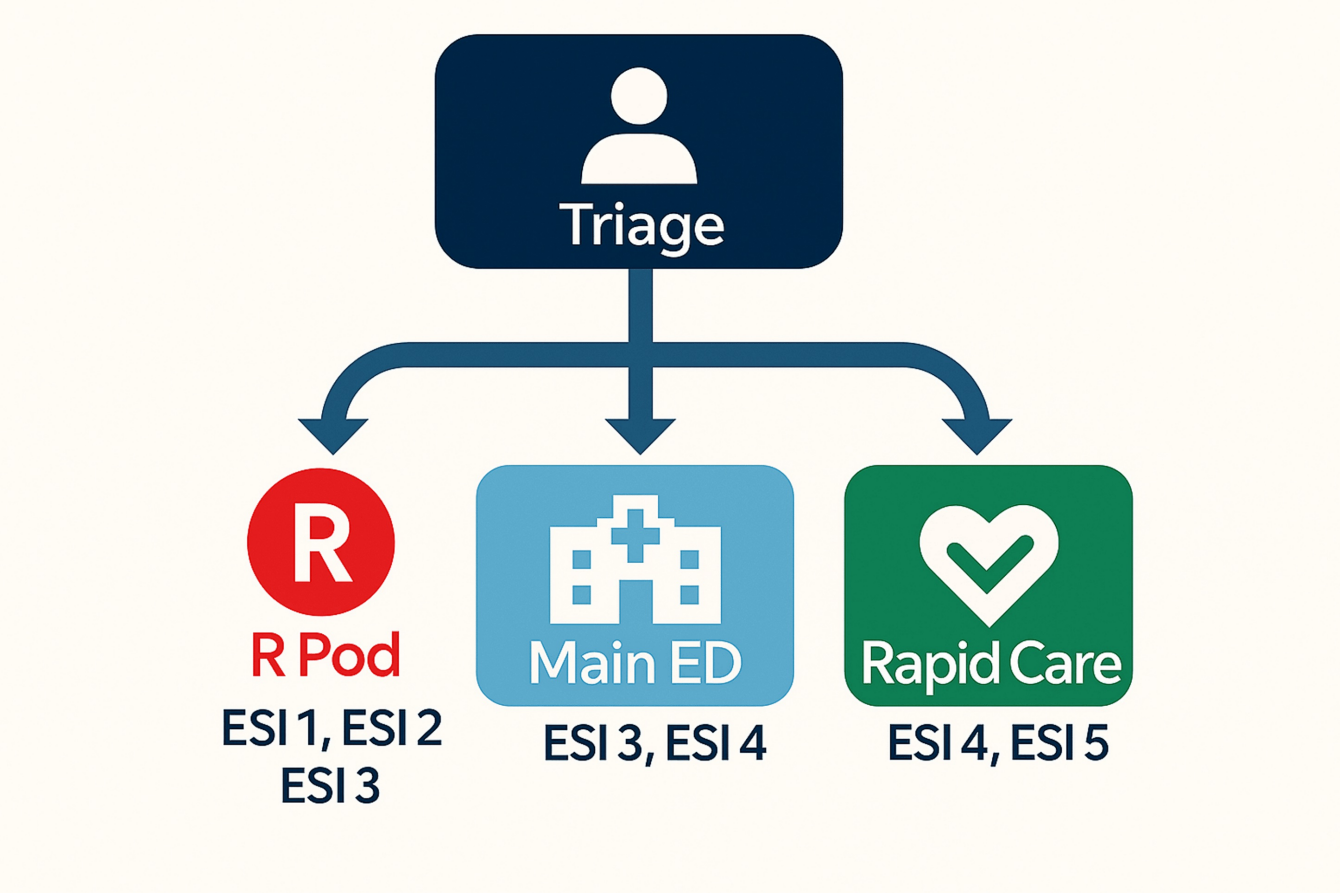
Patient assignment flow. R Pod: Residency Pod; ESI: Emergency Severity Index.

The R pod is located in a separate portion of the emergency department. It is fully staffed 24 hours a day by 2-6 residents, depending on time of day, with supervision by 1-2 attending physicians at all times. Staffing numbers are adjusted to match patient volumes, with increased resident and attending staffing during the hours of 8a to 12a (Figure 1). The R pod is assigned to care for roughly 50% of all patients who present to the ED, with patient selection based on Emergency Severity Index (ESI) version 4 and version 5, perceived patient complexity, and current patient volume. ESI version 5 was adopted by the hospital in 2023. R pod designation is decided by triage staff (Figure 2). The residents working R pod shifts range from training levels PGY-1 - PGY-3. This process is designed so that higher-acuity patients, defined as patients with an ESI score of 1-2, are preferentially evaluated by the R pod, while lower-acuity patients (ESI 4-5) are seen by the non-R pod attending physicians. ESI level 3 patients are selected for R pod evaluation based on overall departmental volume and perceived level of complexity by triage staff.

Study design

This is a retrospective, observational study conducted in a single, community-based emergency department in Modesto, California, with an annual patient census of approximately 70,000. Data were collected from electronic medical records (EMR) and residency scheduling systems for 11 months prior to the implementation of an R pod and for 11 months after implementation (August 1, 2022, to June 30, 2023, and August 1, 2023, to June 30, 2024). The month of July is used for intern orientation, so this month was not included in the data analysis. PGY-1 and PGY-2 classes were used in each data set. No PGY-3 class was available to evaluate until 2023; thus, PGY-3 residents were excluded from this study. There were 8 residents in each class, for a total of 16 residents for each timeframe. All resident shifts were 10 hours. PGY-1 residents worked 18 shifts, while PGY-2 residents worked 17 shifts. Shift totals did not change over the 2 years of this study. The number of patient encounters, the ESI triage ranking of those patients, admit versus discharge status, and the total number of critical procedures were totaled for each class during each time period. In both pre- and post-implementation groups, if more than one resident cared for a particular patient, both residents were credited with the patient encounter, ESI level, and admission for that patient. This was done to account for transition of active patient care during shift change, which can account for a significant amount of clinical time spent. A maximum of 2 residents received credit, and in each case, a care note had been entered in the medical chart indicating transition of active patient care. However, only the resident who performed the procedure was credited for the critical procedure. Critical procedures performed by a resident physician in the emergency department were defined as the following procedures: endotracheal intubation, central venous catheter placement, lumbar puncture, thoracostomy tube placement, procedural sedation, thoracentesis, paracentesis, electrical or medical cardioversion, and orthopedic reduction.

Statistical analysis

The median number of patients seen per month, as well as critical procedures per month, was calculated for each PGY level, as the data were not normally distributed. The Wilcoxon rank-sum test was utilized to determine statistical significance. In addition, ESI levels from 1 to 5 (ESI 1 meaning most acute, ESI 5 being least acute) for each patient encounter were totaled and compared between the pre-implementation and post-implementation periods, as was the total number of patients admitted to an acute care facility. A chi-squared test was utilized to determine differences in proportion among these data. Statistical significance was set at a p-value < 0.05. Analyses were performed using SAS (version 9.4).

Ethical considerations 

The Research Determination Committee for the Kaiser Permanente Northern California Region has determined that the project does not meet the regulatory definition of research involving human subjects per 45 CFR 46.102(d).

## Results

**Figure 3:**
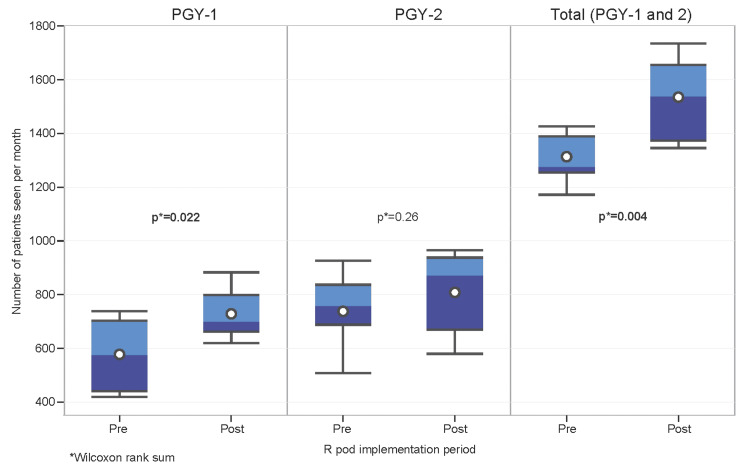
Number of patients seen per month by R Pod implementation period and PGY. R Pod: Residency Pod; PGY: Postgraduate year.

The median number of patient encounters per resident per month increased after the implementation of the R pod (Table 1 and Figure 3). The increase in patient encounters was statistically significant for PGY-1 residents, with the median number of patients per month increasing from 573 to 698 (p=0.022), as well as for the combined PGY-1 and PGY-2 residents, with the median number of patients per month increasing from 1273 to 1537 (p=0.004). Although there was an increase in median patient encounters for the PGY-2 residents from 756 to 870 patients per month, this did not reach statistical significance (p=0.26).

**Table 1:** Median number of patients seen per month by Residency Pod (R Pod) implementation period and postgraduate year (PGY). ^a^Wilcoxon rank-sum test ^b^PGY-1 and PGY-2 each consisted of eight residents. R Pod: Residency Pod; PGY: Postgraduate year.

Resident Year	Pre-R Pod patients, Median (IQR)	Post-R Pod patients, Median (IQR)	W-statistic	p-valueᵃ
PGY-1	573 (439-701)	698 (661-797)	91	0.022
PGY-2	756 (686-835)	870 (667-936)	109	0.26
Total (PGY-1 and PGY-2)ᵇ	1273 (1253-1387)	1537 (1371-1653)	82	0.004

The acuity of patient encounters also increased for residents while working in the R pod (Table 2). Residents cared for an increased proportion of ESI 2 patients. The PGY-1 residents demonstrated an increase in ESI 2 patients from 19.4% to 35.5% (p<0.001), as did the PGY-2 residents, with an increase from 17.3% to 37.2% (p<0.001). The combination of training levels also showed an increase from 18.2% to 36.4% (p<0.001). Data show the percentage of ESI 3 patients, when compared to the total number of all patients evaluated by residents, decreased from 67.8% to 60.3% for the overall cohort, from 68.3% to 61.1% for the PGY-1 residents, and from 67.5% to 59.7% for the PGY-2 cohort. Although the total number of ESI 1 patients increased for each cohort, the proportion of ESI 1 patients in relation to all patients evaluated was not statistically significant (p>0.05) when post-implementation data were compared to pre-implementation data. ESI level 4 and 5 patients significantly decreased (p<0.001) for both cohorts after the implementation of the R pod. The admission rate of patients showed a statistically significant increase for PGY-1 residents from 1105 (17.5%) to 1680 (21.0%) (p<0.001), for PGY-2 residents from 1378 (17.0%) to 2126 (24.0%) (p<0.001), and for both classes from 2483 (17.2%) to 3806 (22.6%) (p<0.001) (Table 3).

**Table 2:** Emergency Severity Index (ESI) level by R Pod implementation period and PGY. ^a^Multiple residents were assigned to some encounters ^b^Chi-square test used to assess overall differences in proportions. Pairwise testing used bootstrap-adjusted p-values to test differences in column percentages for each acuity level ^c^PGY-1 and PGY-2 each consisted of 8 residents. ESI: Emergency Severity Index; R pod: Residency pod; PGY: Post-graduate year.

ESI level by PGY	Pre-R Pod encountersᵃ, N (%)	Post-R Pod encountersᵃ, N (%)	Test statistic (χ²)	df	p-valueᵇ
ESI, PGY-1			757.17	4	<0.001
1 Resuscitative	16 (0.2)	26 (0.3)			0.89
2 Emergent	1225 (19.4)	2835 (35.5)			<0.001
3 Urgent	4327 (68.3)	4885 (61.1)			<0.001
4 Non-Urgent	733 (11.6)	240 (3.0)			<0.001
5 Minor	31 (0.5)	7 (0.1)			<0.001
ESI, PGY-2			1394.49	4	<0.001
1 Resuscitative	28 (0.4)	36 (0.4)			0.95
2 Emergent	1397 (17.3)	3303 (37.2)			<0.001
3 Urgent	5466 (67.5)	5296 (59.7)			<0.001
4 Non-Urgent	1170 (14.4)	227 (2.6)			<0.001
5 Minor	35 (0.4)	10 (0.1)			<0.001
ESI, Total (PGY-1 and PGY-2)ᶜ			2144.04	4	<0.001
1 Resuscitative	44 (0.3)	62 (0.4)			0.85
2 Emergent	2622 (18.2)	6138 (36.4)			<0.001
3 Urgent	9793 (67.8)	10181 (60.3)			<0.001
4 Non-Urgent	1903 (13.2)	467 (2.8)			<0.001
5 Minor	66 (0.5)	17 (0.1)			<0.001

**Table 3:** Hospital admissions by R Pod implementation period and PGY. ^a^Multiple residents were assigned to some encounters ^b^Chi-square test used to test differences in proportions ^c^PGY-1 and PGY-2 each consisted of 8 residents R Pod: Residency Pod; PGY: Postgraduate year; df: Degrees of freedom.

Postgraduate year	Pre-R Pod hospitalization encountersᵃ, N (%)	Post-R Pod hospitalization encountersᵃ, N (%)	Test statistic (χ²)	df	p-valueᵇ
PGY-1	1105 (17.5)	1680 (21.0)	28.71	1	<0.001
PGY-2	1378 (17.0)	2126 (24.0)	124.51	1	<0.001
Total (PGY-1 and PGY-2)ᶜ	2483 (17.2)	3806 (22.6)	139.01	1	<0.001

**Figure 4:**
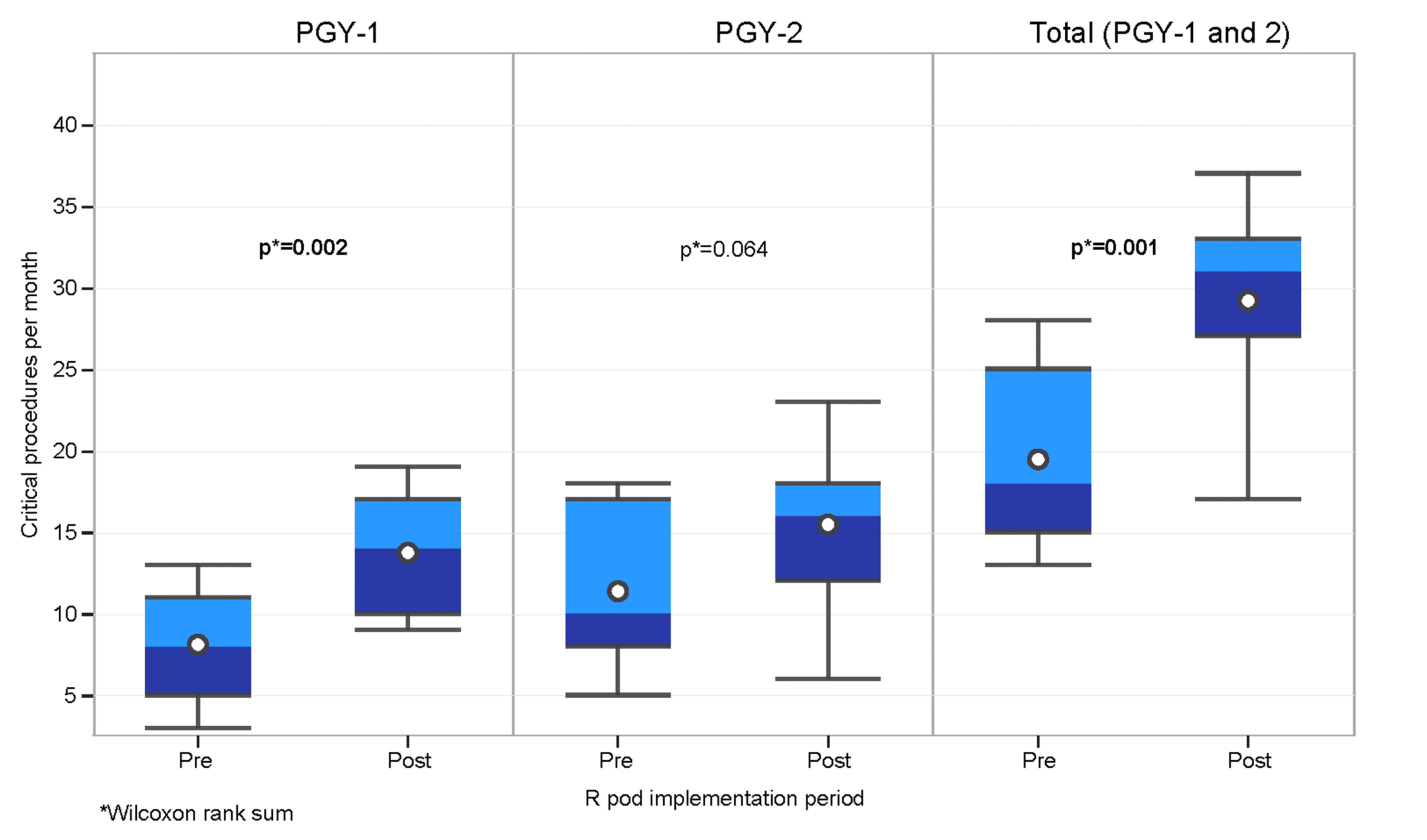
Number of critical procedures per month by R Pod implementation period and PGY. R Pod: Residency Pod; PGY: Postgraduate year.

The median number of critical procedures per month, as defined previously, also showed a significant increase for the PGY-1 residents (p=0.002) and for the combined PGY-1 and PGY-2 groups (p=0.001). The PGY-2 cohort also had an increased number of critical procedures, but this did not reach statistical significance (p=0.064) (Table 4 and Figure 4).

**Table 4:** Median number of critical procedures per month by R Pod implementation period and PGY. ^a^Wilcoxon rank-sum test ^b^PGY-1 and PGY-2 each consisted of 8 residents R Pod: Residency Pod; PGY: Postgraduate year.

Postgraduate year	Pre-R Pod critical procedures, Median (IQR)	Post-R Pod critical procedures, Median (IQR)	W-statistic	p-valueᵃ
PGY-1	8 (5-11)	14 (10-17)	80	0.002
PGY-2	10 (8-17)	16 (12-18)	98	0.064
Total (PGY-1 and PGY-2)ᵇ	18 (15-25)	31 (27-33)	79	0.001

## Discussion

The implementation of the R pod system within a community-based EM training program demonstrated a significant association with patient volume managed by EM residents, the acuity level of those patients, and procedural opportunities for residents. Our analysis revealed a significant increase in the number of patients seen by residents and a higher acuity level of those patients following R pod implementation, suggesting that this model may contribute to an increased number of resident learning opportunities and clinical exposure. Furthermore, residents were able to complete a significantly higher number of critical procedures, which also suggests an enhanced environment for clinical learning opportunities.

One of the primary objectives of the R pod was to concentrate critically ill or procedurally complex patients under the care of residents, allowing for richer clinical experiences. This structured exposure appears to have translated into increased patient volume, procedural volume, and exposure to high-acuity patients, particularly among junior residents. The largest benefit was seen among patients with an ESI-2 acuity level, as both PGY-1 and PGY-2 cohorts demonstrated significantly increased exposure to this patient demographic. While both PGY-1 and PGY-2 cohorts were exposed to an overall greater number of patients and procedures after R pod implementation, the results indicate that the PGY-1 cohort experienced a statistically significant benefit from the R pod design. We suspect that one factor contributing to this finding was that PGY-2 residents were more likely to give procedures to the PGY-1 cohort and assume a more supervisory role. We also suspect that because the acuity of the PGY-2 cohort increased, it may have decreased the ability of these residents to care for significantly higher volumes of patients when compared to the previous system of patient assignment. However, no analysis was performed to specifically address this incongruency. Our findings are consistent with previous literature that highlights the value of early and structured exposure to high-acuity cases in fostering procedural confidence and skill acquisition [7,9]. Furthermore, we suspect the hierarchical design of the R pod may have allowed for meaningful peer-to-peer learning, as senior residents played a supervisory role, thereby reinforcing their knowledge while enhancing the educational experience for junior residents. However, this was not directly evaluated and should be investigated in future studies.

Prior to the R pod system, resident shifts operated on a traditional 1-on-1 model with attending physicians, which, while valuable, often limited the variety and acuity of cases available to each resident, based on compiled feedback. The R pod’s approach to patient assignment, based on acuity and procedural potential, appears to have mitigated this limitation by streamlining the potential educational value of each shift through higher-acuity patients, increased patient volumes, and increased procedural opportunities. Of note, during the R pod implementation process, concern was raised during PEC meetings that residents may not be exposed to enough ESI 4 and ESI 5 patients during their training. However, residents in our program work dedicated rapid care shifts, which are designed to care for only ESI 4 and ESI 5 patients.

Importantly, while residents generally improve in efficiency over time due to increased familiarity with workflows and clinical decision-making [10], our study design controlled for this natural progression by comparing similar timeframes and accounting for resident training level. Thus, we believe that the observed increases in performance are likely attributable to the structural changes introduced by the R pod system, rather than solely the passage of time or cumulative experience. However, this study is limited by a small sample size within a single medical center.

From an operational standpoint, the R pod model may also offer benefits beyond providing increased clinical opportunities for residents. Concentrating complex cases among a dedicated team may enhance consistency in care delivery, streamline attending oversight, and improve interprofessional collaboration within a focused physical space. While not directly addressed by our study, this is supported by studies indicating improved ED performance metrics with early patient assignment to dedicated teams [3,8].

Our study possesses several limitations. The comparative nature of this study design lacks a control group to determine direct causality, and as a single-institution, retrospective analysis with a small sample size, our findings may not be generalizable to other EM programs with different structures, resources, or patient populations. Although all ESI 1 and 2 patients were routinely assigned to the R pod, there was some subjectivity regarding which ESI 3 patients were assigned to the R pod, which may also contribute to variability of results in other settings. Additionally, while we accounted for confounders such as overall clinical time spent caring for patients and level of training, other unmeasured factors such as faculty teaching style, resident motivation, or seasonal variation in ED volume may have the potential to influence outcomes. Individual resident motivation, efficiency on shift, and overall clinical competency may also be confounding factors, exacerbated by a small sample size, particularly since one cohort was observed as a PGY-1 class and again as a PGY-2 class. Furthermore, we attempted to account for transitions of care by crediting two residents for one patient if medical records indicated resident involvement. Although we applied this same method to both pre- and post-implementation groups, there is potential for artificial inflation in total numbers. However, we felt it necessary to attempt to capture the patient care delivery and clinical educational opportunities that accompany the continuous nature of EM. Future studies could include prospective, multi-site evaluations with larger cohorts and more granular qualitative data to assess resident perceptions of educational value and preparedness for independent practice. In addition, it would be beneficial to observe the impact of the R pod system over a longer timeframe and to directly assess educational value in the form of competency-based outcomes.

## Conclusions

In conclusion, the introduction of the R pod system was associated with a significant increase in patient encounters, patient acuity, and procedural volume among EM residents. This model appears to contribute to an increase in surrogate markers of resident educational opportunities by prioritizing high-yield clinical experiences and structured supervision. This model may be particularly useful for smaller, community-based educational programs that do not have a complement of residents large enough to evaluate all patients presenting to the ED. As EM residency programs seek to optimize training in increasingly complex clinical environments, the R pod system is a promising approach to balancing educational depth with operational efficiency.
